# Transcript-Targeted Therapy Based on RNA Interference and Antisense Oligonucleotides: Current Applications and Novel Molecular Targets

**DOI:** 10.3390/ijms23168875

**Published:** 2022-08-09

**Authors:** Vincenza Barresi, Camillo Musmeci, Alessandro Rinaldi, Daniele Filippo Condorelli

**Affiliations:** Section of Medical Biochemistry, Department of Biomedical and Biotechnological Sciences, University of Catania, 95123 Catania, Italy

**Keywords:** antisense oligonucleotide (ASO), RNA interference (RNAi), genetic diseases, splicing modification, cancer, transcript-targeted therapy

## Abstract

The development of novel target therapies based on the use of RNA interference (RNAi) and antisense oligonucleotides (ASOs) is growing in an exponential way, challenging the chance for the treatment of the genetic diseases and cancer by hitting selectively targeted RNA in a sequence-dependent manner. Multiple opportunities are taking shape, able to remove defective protein by silencing RNA (e.g., Inclisiran targets mRNA of protein PCSK9, permitting a longer half-life of LDL receptors in heterozygous familial hypercholesteremia), by arresting mRNA translation (i.e., Fomivirsen that binds to UL123-RNA and blocks the translation into IE2 protein in CMV-retinitis), or by reactivating modified functional protein (e.g., Eteplirsen able to restore a functional shorter dystrophin by skipping the exon 51 in Duchenne muscular dystrophy) or a not very functional protein. In this last case, the use of ASOs permits modifying the expression of specific proteins by modulating splicing of specific pre-RNAs (e.g., Nusinersen acts on the splicing of exon 7 in SMN2 mRNA normally not expressed; it is used for spinal muscular atrophy) or by downregulation of transcript levels (e.g., Inotersen acts on the transthryretin mRNA to reduce its expression; it is prescribed for the treatment of hereditary transthyretin amyloidosis) in order to restore the biochemical/physiological condition and ameliorate quality of life. In the era of precision medicine, recently, an experimental splice-modulating antisense oligonucleotide, Milasen, was designed and used to treat an 8-year-old girl affected by a rare, fatal, progressive form of neurodegenerative disease leading to death during adolescence. In this review, we summarize the main transcriptional therapeutic drugs approved to date for the treatment of genetic diseases by principal regulatory government agencies and recent clinical trials aimed at the treatment of cancer. Their mechanism of action, chemical structure, administration, and biomedical performance are predominantly discussed.

## 1. Introduction

“Transcript-targeted therapy” can be defined as any molecular treatment able to modify transcriptionally or post-transcriptionally the levels of coding and noncoding RNAs in order to obtain a therapeutic advantage.

**Figure 1:**
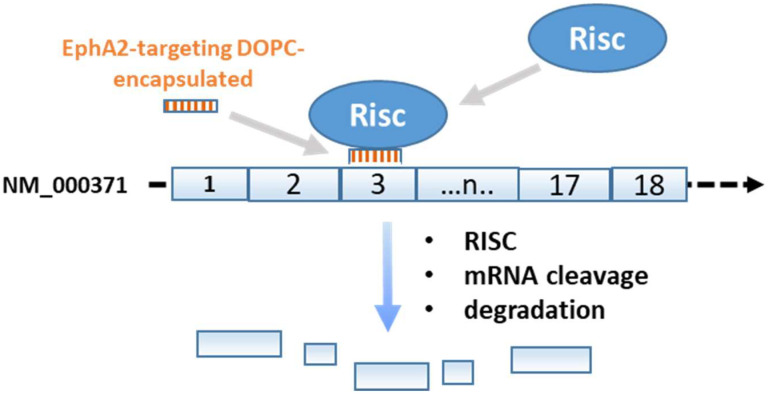
siRNA treatment to silence the ephrin-A receptor 2 (EphA2). The gene is located on chromosome 1p36.13; exons, 18; NM_004431; NP_004422. A detailed description is reported in the text.

**Figure 2:**
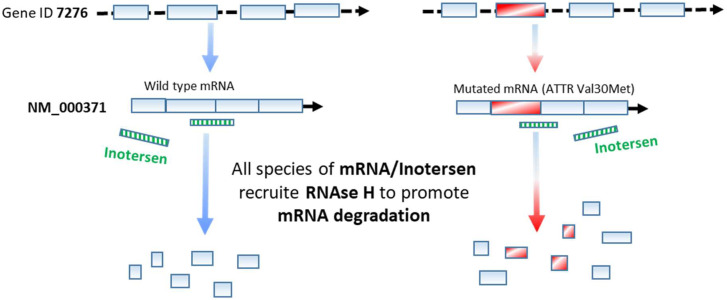
Downregulation of human TTR transthyretin (hTTR) transcripts mediated by ASO Inotersen. The gene (ID 7276) is located on chromosome 18q12.1; exons, 4; NM_000371; NP_000362. A detailed description is reported in the text.

**Figure 3:**
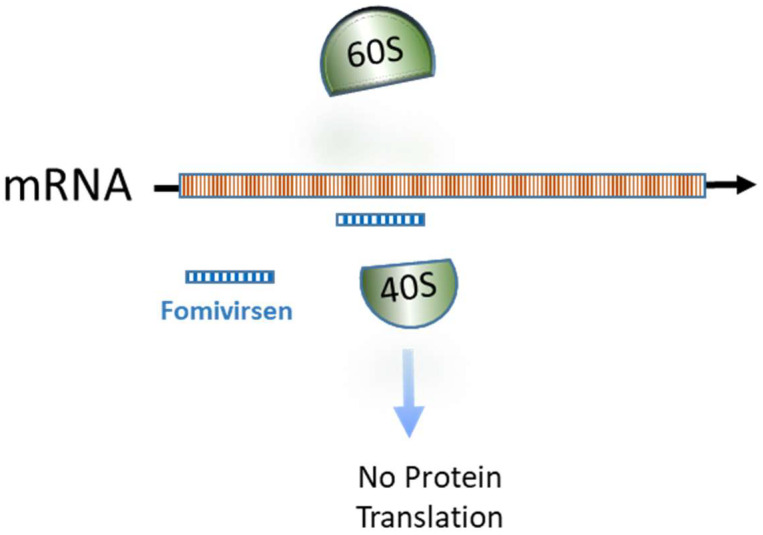
mRNA/ASO–Fomivirsen interaction prevents RNA loading in ribosome and blocks mRNA translation into protein IE2 in the cytoplasm. A detailed description is reported in the text.

**Figure 4:**
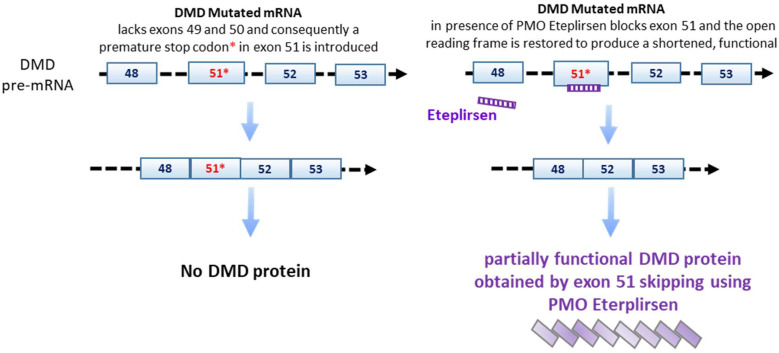
Exon skipping through PMO Eteplirsen to restore Dystrophin (DMD) open reading frame. The gene (ID 1756) is located on Chromosome X. ID; exons, 89; NM_004006; NP_003997. A detailed description is reported in the text.

**Figure 5:**
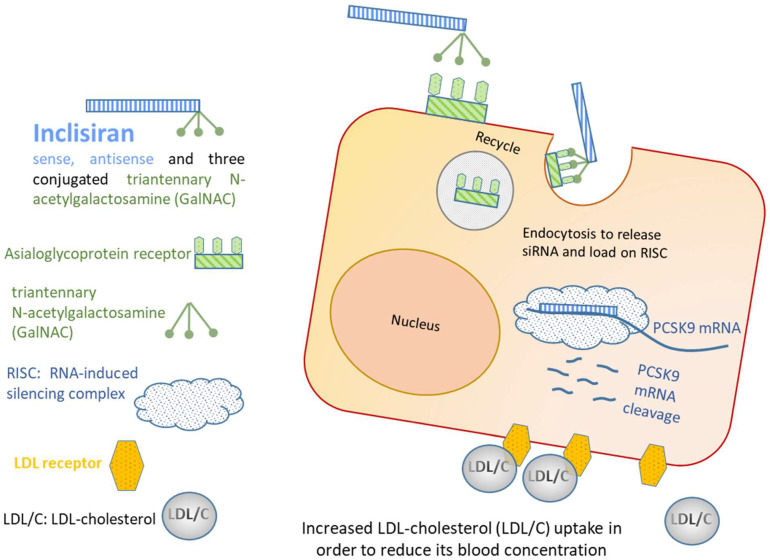
Inclisiran recognizes PCSK9 mRNA to promote its degradation and to reduce protein translation; in this way, Inclisiran prolongs the half-life of LDL receptors that can continue to capture LDL-cholesterol, determining its blood reduction. A detailed description is reported in the text.

The more common tools for transcriptional regulatory therapy are based on RNA interference (RNAi) or antisense oligonucleotides (ASOs) and their basic mechanisms are summarized in Figure 1, Figure 2, Figure 3, Figure 4 and Figure 5. Exogenous messenger RNAs (mRNAs) as therapeutics and the Genome Editing tools, primarily based on the use of clustered regularly interspaced short palindromic repeats and CRISPR-associated protein 9 (CRISPR-Cas9) deserve to be analyzed and discussed in another review for the large amount of data collected in the last decade about them. In this review, we describe the molecular targets and the pharmaceutical formulations of ASO- and siRNA-based therapeutics that have been approved for human clinical use or have been investigated in the last 6 years in human clinical trials.

## 2. siRNAs and ASOs

“RNA interference or RNA silencing (RNAi)” is a process in which double-stranded RNA (dsRNA) is processed into short interfering RNAs (siRNAs) that downregulate gene expression by degradation of targeted mRNA molecules (Figure 1). The history of RNAi research began in the early 1990s when a number of scientists, through their studies on plants and fungi, independently observed that RNA molecules in some particular conditions could inhibit gene expression at the post-transcriptional level [1,2]. These phenomena, initially known as “Post-Transcriptional Gene Silencing” (PTGS), quelling, and cosuppression, were not fully understood in their mechanism until 1998 when Andrew Fire and Craig Mello demonstrated that double-stranded RNAs (dsRNA) were the causative agents [3]. This discovery made possible the unification of the previous observations in a single-cellular mechanism that they called RNA interference (RNAi, Nobel Prize in Physiology or Medicine, 2006). In 2001, Elbashir and Caplen independently [4,5] found out that 21–22 nucleotides could induce RNAi in mammalian cells without triggering nonspecific interferon responses, normally induced by >30 nt dsRNA, and soon these small interfering RNAs (siRNAs) were identified as a therapeutic tool for the treatment of numerous diseases such as cancer, viral infections, and neurodegenerative diseases. In the cytoplasm, siRNAs are loaded into the “RNA-induced silencing complex” (RISC); generally, only one strand (guide strand) is incorporated into the RISC, the other strand (passenger strand) is normally discarded and degraded. After loading into the RISC, the guide strand targets the transcript with a total complementarity and triggers an endonucleolytic cleavage (mediated by a RISC-associated protein called “Argonaute-2” or “Ago2”), which induces its degradation and inhibits the protein translation [6]. A simplified schematic draw is reported in Figure 1. Two types of small RNA molecules are inductors of the RNAi pathway: endogenous “microRNA” (miRNA), produced by the own cellular genome, and exogenous “small interfering RNA” (siRNA), derived from extracellular genomes such as virus or artificially introduced for experimental or therapeutically purposes. Unlike siRNAs, miRNAs recognize mRNA targets with an imperfect complementarity and, also in this case, typically silence genes by repression of translation [7].

“Antisense Oligonucleotides” (ASOs) are short-length single sequences of deoxynucleotides, 12–28 bases, that are synthesized to be complementary to a sequence of mRNA or pre-mRNA to generate a DNA/RNA heteroduplex; in this way, they can regulate the target expression. For the first time, Zamecnik and Stephenson used ASO to inhibit Rous sarcoma virus (RSV) cycle [8]. Generally, the action of ASO can be mediated through different mechanisms:

- A “RNAse-dependent mechanism” that takes place in the nucleus and is mediated by the enzyme “RNAse H”, able to degrade the RNA strand of RNA/DNA duplex (Figure 2).
- “RNAse-independent mechanisms” that take place in the cytoplasm; in this case, the ASO can act through different paths:
  a. One is dependent from the interaction between ASO and transcript, thus preventing RNA loading on the ribosome and arresting mRNA translation (Figure 3) [9];
  b. Another mechanism is operating through the splicing process [10]—that is, modifying the open reading frame. This mechanism could have wide applications such as in ataxia telangiectasia or Duchenne muscular dystrophy (Figure 4) [11].

It is evident that ASOs need high affinity and stability with the target transcripts and they must resist nuclease activity; for this reason, different chemical modifications of ASOs have been performed to preserve the sequence integrity and to allow them to reach their effect. On the basis of their chemical modifications, they are classified in first, second, and third generation (Table 1).

“First-generation ASOs” are characterized by a modification in the phosphate backbone—in which a nonbridging oxygen atom is substituted by a sulfur atom—and are called “phosphorothioate ASOs” (PS-ASOs). The first developed PS-ASO is the “Fomivirsen”, used in the treatment of Cytomegalovirus (CMV) retinitis [12,13,14]. The phosphorothioate sequence of twenty-one nucleotides in length (5′-GCGTTTGCTCTTCTTCTTGCG-3′) makes ASOs more stable and allows a better uptake without altering their RNAse-dependent activity or their binding to mRNA [15]. PS-ASO Formivirsen binds to UL123 transcripts and inhibits the translation into IE2 protein (Figure 3).

“Second-generation ASOs” have a modification at the 2′ position of ribose, such as the 2-O-methyl (2-OME) and the 2-O-methoxyethyl (2-MOE). This ASO cannot induce RNAse H activity, so it is possible to create a structure with central PS-oligonucleotides between these chemical groups; this structure is also called “gapmer”. The molecule is an ASO with considerable affinity and resistance to nucleases. Another modification, the 2-O-methylcarbamoylethyl (2-O-MCE), yields ASOs with similar RNAse-inducing activity but less hepatotoxicity [16]. An example of second-generation ASO is “Inotersen”, designed and approved for the treatment of Hereditary Transthyretin Amyloidosis (hATTR, Figure 2, Table 1; see also Section 2).

“Third-generation ASOs” are characterized by chemical modifications to the monosaccharide. One group of third-generation ASOs are “phosphorodiamidate morpholino oligonucleotides” (PMOs), containing a morpholino ring and nonionic linkages [17]. The other ASOs of this generation are “peptide nucleic acids” (PNAs). Both PNAs and PMOs are characterized by a better stability and a RNAse-H-independent mechanism. PNAs are oligodeoxynucleotide analogs in which the deoxyribose phosphodiester backbone is replaced by a pseudo-peptide polyamide backbone [18]. They can affect gene expression inhibiting transcription by binding to the DNA or translation by binding to the mRNA. To date, no PNAs have been approved for the treatment of genetic diseases and cancer except for the detection of SARS-CoV-2 nucleic acid in biological samples (FDA approval May 2021, https://www.fda.gov/, accessed on 30 April 2022).

Another important issue is the delivery of ASOs to their targets [19,20] and the mechanisms of cellular recognition and internalization [21]. Intraocular, intravenous, intrathecal, and subcutaneous administrations of ASOs are adopted to reach the target cellular districts in association with various delivery systems, such as the conjugation with cellular-penetrating peptides (CPP) [18] or the use of liposomal structure [20]. Indeed, there are a lot of receptors expressed on the cell membrane that can mediate the uptake of ASOs [21]. Moreover, studies about ASO toxicity are important in order to estimate the balance between therapeutic and toxic effects. According to the pharmacokinetic profile, the liver and kidney are the main tissues where we can observe high levels of ASOs. In particular, the kidney explicates a major part of ASOs’ elimination. So, the main adverse drug reactions (ADRs) involve nephrotoxicity and hepatotoxicity. Other ADRs are represented by mild hyperglycemia, autoimmune reactions, activation of the complement, and hypotension [22,23].

In 1998, the FDA approved the first ASO drug called “fomivirsen” (trade name Vitravene) followed by others such as “mipomersen”, “eteplirsen”, and “nusinersen”, which were authorized for clinical use (Table 1, Figure 1, Figure 2, Figure 3, Figure 4 and Figure 5) [24].

**Table 1:** List of main ASOs classified on the basis of their chemical modifications in first, second, and third generation.

		Name (Trade Name)	Sequence and Mechanism of Action	Year of Approval	Disease Treatment	Delivery Route
First generation	Phosphotionate link group phosphate backbone in which a nonbridging oxygen atom is substituted by a sulfur atom	Fomivirsen (Vitravene)	5′-dGdCdGdTdTdTdGdCdTdCdTdTdCdTdTdCdTdTdGdCdG-3′ ASO binds to UL123-RNA and blocks the translation into IE2 protein	1998 (FDA), 1999 (EMA)	CMV-retinitis	Intraocular
Second generation	Gapmer structure Containing a modification at the 2′ position of ribose, such as the 2-O-methyl (2-OME) and the 2-O-methoxyethil (2-MOE).	Inotersen (Tegsedi)	5′TCTTG GTTACATGAA ATCCC methylated C is indicated in bold, underlined bases are 2-O-MOE-modified riboses	2018 (FDA), 2018 (EMA)	hATTR	Subcutaneous
	Gapmer structure phosphate backbone in which a non-bridging oxygen atom is substituted by a sulfur atom and modification at the 2′ position of ribose (2-MOE)	Mipomersen (Kynamro)	5′GCCUC AGTCTGCTTC GCACC Underlined bases are 2-O-MOE nucleosides and all other residues are 2–deoxynucleosides. Cytosine (C) and uracil (U) bases are modified at the 5 position with a methyl group.	2013 (FDA)	familial hyper-cholesterolemia	subcutaneous administration
Second generation	Gapmer structure Modified 2′-O-2- Methoxyethyl (2-MOE) phosphorothioate antisense oligonucleotide	Nusinersen (Spinraza)	5′MeUMeCAMeCMeUMeUMeUMeCAMeUAAMeUGMeCMeUGG	2016 (FDA), 2017 (EMA)	SMA	intrathecal injection
Third generation	Peptide Nucleic Acids (PNAs, Nielsen, 2004)	--- COVID-19 RT-PCR Peptide Nucleic Acid (PNA) Kit	---	No approved for disease and cancer treatment FDA (2021)	---	--- To detection SARS-CoV-2 naso/oropharyngeal or anterior or nasal specimens
Third generation	Phosphorodiamidate Morpholino Oligonucleotides (PMOs)	Eteplirsen (Exondys 51)	5′-CTCCAACATCAAGGAAGATGGCATTTCTAG-3′	2016 (FDA)	DMD disease	Intravenous

## 3. Transcript-Targeted Therapies for Genetic Diseases

The first transcript-targeted therapies authorized for use in clinical practice are focused against genetic diseases. These therapies, based on RNAi or ASOs, can be distinguished between **those acting through regulation of transcript levels (1)** and **those modifying the splicing of specific pre-RNAs (2)**.

*(1). Downregulation of transcript levels by RNAi-based drugs (Patisiran, Teprasiran, QPI-1007, Inclisiran, Tofersen) by the ASO-based drug (Inotersen) and second-generation ASO-based drug (Volanesorsen/Waylivra).*

**(A) Hereditary Transthyretin Amyloidosis (hATTR)** is a rare autosomal dominant, multisystemic, progressive disease caused by more than 120 mutations in the gene encoding transthyretin (TTR) located on chromosome 18q12.1. The protein TTR binds and transports thyroxine and retinol. The most common mutation is a single-nucleotide substitution that causes the substitution of Valine in position 30 with Methionine (ATTR Val30Met) (Figure 2). The association of altered TTR makes amyloid fibrils and leads to the formation of amyloid plaques in numerous organs such as the peripheral nerves, heart, kidneys, and gastrointestinal tract. The following main clinical manifestations are polyneuropathy, characterized by sensorimotor and autonomic disturbances, and cardiomyopathy, which mainly occurs with arrhythmias and heart failure [25,26]. hATTR is a life-threatening disease with median survival between 5 and 15 years from diagnosis. The poor prognosis and short life expectancy of this disease are in a large part due to the poor effectiveness of the current treatment options (liver transplantation and transthyretin stabilization with tafamidis or diflunisal) in keeping disease progression under control [27].

An alternative therapeutic target is represented by the reduction in the circulating level of TTR, thus decreasing the amount of proteins that can form the amyloid fibrils. Two drugs have been designed based on this mechanism of action: “inotersen”, an ASO-based drug, and “patisiran”, a RNAi-based drug. These drugs are useful alternatives to other classical treatment such as liver transplantation and TTR stabilizers.

“Inotersen” (trade name Tegsedi produced by Akcea Therapeutics), a second-generation ASO containing a 2′-O-methoxyethyl modification, is designed to target TTR mRNA to inhibit hepatic TTR production and, consequently, amyloid fibrils and plaques (Table 1, Figure 2). In a clinical trial (NEURO-TTR ClinicalTrials.gov number: NCT01737398, https://clinicaltrials.gov/ct2/show/NCT01737398, accessed on 30 April 2022), inotersen was administered by subcutaneous injections, three times a week for the first week followed by once a week administration for 64 weeks. Patients that received inotersen obtained an improvement of their life’s quality. However, they showed different ADRs such as glomerulonephritis, thrombocytopenia, and death [28].

“Patisiran” siRNA (ALN-18328) downregulates wild-type and mutant TTR protein expression in hepatocytes, targeting the 3′UTR region of the TTR mRNA and mediating its degradation (Table 2). Patisiran siRNA is delivered by a “second-generation” pegylated lipid nanoparticle (LNP) composed of cholesterol, the polar phospholipid Distearoylphosphatidylcholine (DSPC), an ionizable aminolipid (6Z,9Z,28Z,31Z)-heptatriacont-6,9,28,31-tetraene-19-yl 4-(dimethylamino)butanoate (DLin-MC3-DMA), and a pegylated lipid (PEG2000-C-DMG). In the bloodstream, apolipoprotein E (ApoE) replaces PEG2000-C-DMG and is incorporated into the LNP lipid matrix. In the liver, ApoE-covered LNPs are internalized by endocytosis and siRNA is then released into the cytosol where it can perform its pharmacological action [29].

APOLLO, a phase III, double-blind, placebo-controlled clinical trial for patisiran, began in December 2013. This clinical trial recruited 225 patients with hATTR aggravated by polyneuropathy: 77 and 148 patients were assigned, respectively, to the placebo and the Patisiran arms. All of them, after receiving premedication to reduce the risk of infusion-related reactions, were treated with Patisiran (0.3 mg per kg) or placebo intravenously once every 3 weeks for 18 months. The trial demonstrated that the Patisiran group accomplished > 70% reduction in transthyretin from baseline, 56% improved mNIS +7 (modified neuropathy index score +7) versus 4% in the placebo group, and 51% improved quality of life (statistically tested by Norfolk QOL-DN questionnaire) versus 10% in the placebo arm. Finally, the trial showed no risk of death associated with Patisiran treatment and similar incidences of both severe and serious adverse events in the two study arms [30] (ClinicalTrials.gov Identifier: NCT01960348, https://clinicaltrials.gov/ct2/show/NCT01960348, accessed on 30 April 2022).

Efficacies of patisiran and its direct competitor, the ASO-based drug inotersen, were tested in two randomized, double-blind, controlled trials implemented by Adams et al. (2018) [30] and Benson et al. (2018) [28]. These trials showed that Patisiran achieves better results than Inotersen both in terms of efficacy (serum levels of transthyretin reduced by 81% with Patisiran versus 71% with Inotersen) and safety (Inotersen systematically causes thrombocytopenia). Despite these results, the efficacy of both drugs over periods longer than 18 months are under investigation [31].

In June 2022, FDA approved “vutrisiran” for the treatment of hATTR Amyloidosis with polyneuropathy [32]. Vutrisiran (or AMVUTTRA) is a chemically modified double-stranded siRNA that hits mutant and wild-type TTR mRNA and is covalently linked to a tail containing three *N*-acetylgalactosamine (GalNAc) to enable delivery of the siRNA to hepatocytes in order to cause degradation of mutant and wild-type TTR transcript through RNA interference. In this way, a reduction in serum TTR protein and TTR protein deposits in tissues is determined. Clinical studies suggest subcutaneous injection (25 mg) once every 3 months.

**(B) Familial chylomicronemia syndrome (FCS)** is a syndrome characterized by high levels of chylomicrons due to autosomal recessive mutation of the lipoprotein lipase (LPL) gene located on chromosome 8p21.3 or the Apolipoprotein C2 (APOC2) gene located on chromosome 19q13.32. LPL is an enzyme expressed on the cell membrane that hydrolyzes triglycerides, contained in VLDL and chylomicrons, into glycerol and fatty acids; APOC2 is an apoprotein that acts as a cofactor of LPL. FCS can determine some complications such as lipemia retinalis, acute pancreatitis, xanthomas, and diabetes. To treat this syndrome, it is possible to inhibit the expression of APOC3, because it is an inhibitor of LPL, in order to improve the activity of LPL [33]. “Volanesorsen” is the first drug based on a second-generation ASO, containing 2′-O-methoxyethyl modification, that applies this mechanism: in fact, it has as target the APOC3′s mRNA. A study realized by treating 57 patients with volanesorsen administered by subcutaneous injection has shown the efficacy of this drug to reduce the level of triglycerides and to increase the level of HDL [34]. On the other hand, an obstacle is represented by the adverse drug reactions linked to the use of the drug—in particular, the thrombocytopenia. On 28 February 2019, EMA (European Medicine Agency) approved the use of “waylivra”, which is the trade name of volanesorsen produced by Akcea Therapeutics Ireland Ltd. for the treatment of patients affected by FCS. The use of this drug is indicated in patients with a high risk of pancreatitis that show low response to the other classical drugs and diets.

**(C) Delayed graft function (DGF)** is one of the most serious manifestations of acute kidney injury (AKI) that occurs after kidney transplantation. Although there is no unique definition of this condition, in 69% of studies (reviewed between 1984 and 2007) it is defined as the use of dialysis within 7 days of the transplant [35]. Pathogenesis of DGF, which is not yet completely understood, is due to innate and adaptive immune response and, above all, acute ischemia-reperfusion injury (IRI) [36]. Recent research demonstrated the key role played by the increased expression of proapoptotic protein p53 in renal tubular cells as a result of DGF (and more generally of AKI) [37]. “Teprasiran” (QPI-1002), developed by Quark Pharmaceuticals, is a synthetic and chemically modified siRNA drug that temporarily downregulates the expression of proapoptotic protein p53, protecting kidneys from programmed cell death induced by IRI and preserving tissue and organ integrity (Table 2). A phase III pivotal trial showed teprasiran efficacy in the treatment of DGF following kidney transplantation; for this reason, it was designated as an orphan drug for prophylaxis of DGF (ClinicalTrials.gov Identifier: NCT02610296; https://clinicaltrials.gov/ct2/show/NCT02610296, accessed on 30 April 2022). Teprasiran has also achieved positive therapeutic results for prevention of AKI in high-risk patients undergoing cardiovascular surgery, as shown by a multicenter, double-blind, placebo-controlled phase II trial (ClinicalTrials.gov Identifier: NCT02610283; https://clinicaltrials.gov/ct2/show/NCT02610283, accessed on 30 April 2022). Recently, Thielmann et al. (2021) [38] reported that the incidence, severity, and interval of early AKI in high-risk patients undergoing cardiac surgery were significantly reduced after teprasiran treatment. A total of 1043 participants with a major adverse kidney event were enrolled in a randomized, double-blind, placebo-controlled, phase 3 study in order to evaluate the efficacy and safety of teprasiran for the prevention of major adverse kidney events in subjects at high risk for AKI (ClinicalTrials.gov Identifier: NCT03510897, https://clinicaltrials.gov/ct2/show/NCT03510897, accessed on 30 April 2022).

**(D) Nonarteritic ischemic optic neuropathy (NAION)** is a common optic neuropathy caused by infarction of the short posterior ciliary arteries that supply the anterior portion of the optic nerve head. This infarct event induces optic nerve axonal edema and optic disc compartment syndrome, leading to an acute, unilateral, painless vision loss. Although the pathogenesis of NAION is not yet fully understood in detail, it is presumed to be a multifactorial disease caused by a transient disturbance in the circulation of optic nerve head, probably due to generalized hypoperfusion, vasospasm, or thrombosis [39]. Ocular neuroprotection plays a key role in the treatment of NAION and, in particular, the preservation of retinal ganglion cells (RGC): it has been shown that optic nerve injury induces apoptosis of this cellular population through the activation of the proapoptotic protein Caspase-2 [40]. “QPI-1007”, developed by Quark Pharmaceuticals, is a synthetic and chemically modified siRNA drug that inhibits the expression of Caspase-2 (Table 2). QPI-1007 demonstrated therapeutic efficacy both in animal models of acute and chronic ocular neurodegeneration (inducing a significant neuroprotective effect) and in Phase I/II trials in patients with NAION, where it has been observed that a single intravitreal injection of QPI-1007 slows down or even blocks the visual deterioration that is characteristic of this disease (ClinicalTrials.gov Identifier: NCT01064505; https://clinicaltrials.gov/ct2/show/NCT01064505, accessed on 30 April 2022).

**(E)Familial hypercholesteremia (FH)** is an inherited disease in which LDL cholesterol (bad) in blood is over 190 mg/dL in adults. Untreated FH increases risk for developing coronary artery disease or leads to heart attacks. “Mipomersen” is a PS-ASO that specifically binds to Apo B-100 mRNA blocking the translation. The drug is approved to treat homozygous FH and is administered by subcutaneous injection [41,42,43].

Recently, at the end of last year, FDA approved Novartis Leqvio^®^ (Inclisiran)—a synthetic, chemically modified, double-stranded siRNA able to lower cholesterol and keep it low with two doses a year. This adjuvant drug has been approved for the patients affected by heterozygous familial hypercholesteremia and atherosclerotic cardiovascular disease. The patients treated with statin therapy require further reduction in uncontrolled LDL-cholesterol levels. Three subcutaneous doses of Inclisiran (284 mg) at months 0, 3, and 6 are given by a healthcare professional [44]. siRNA drug reaches the target hepatic organ through circulation. siRNA contains 32 ribonucleotides chemically modified with 2-O-methyl-ribonucleotide (2′-O-methyl), 11 modified with 2′-fluoro-ribonucleotide (2′-fluoro), and one 2′-deoxy-ribonucleotide. In addition, two phosphorothioate groups are added to the 5′ end of the sense and both the 5′ and 3′ ends of the antisense strands. In the liver, the siRNA drug conjugated with triantennary *N*-acetylgalactosamine sugars is rapidly internalized by the abundant expression of membrane asialoglycoprotein receptors (ASGPR). In the hepatocyte, the antisense strand in the RISC complex binds to the complementary sequence in its target mRNA of protein PCSK9 to promote mRNA degradation and to reduce its translation (Figure 5). PCSK9 is the protein that binds low-density lipoprotein cholesterol receptors (LDLR) to trigger its degradation by proteosome; in this way, the reduced expression of PCSK9 minimizes their targeting for degradation and permits a longer half-life of LDL receptors on the membrane of hepatocytes available to bind and take in LDL-Cholesterol (LDL/C), resulting in its blood reduction (Figure 5). Adverse effects in a limited number of patients encompass development of antidrug antibodies to Inclisiran/Leqvio, bronchitis, myalgia, headache, fatigue, nasopharyngitis, back pain, hypertension, diarrhea, and dizziness. The clinical trials for this promising therapy have been started in 2014 (2014–2018, Phase I/II trials, https://clinicaltrials.gov/ct2/show/NCT02314442, accessed on 30 April 2022; ORION-1, https://clinicaltrials.gov/ct2/show/NCT02597127, accessed on 30 April 2022; ORION-2, https://clinicaltrials.gov/ct2/show/NCT02963311, accessed on 30 April 2022; 2017–2019, Phase III trials, ORION-9, https://clinicaltrials.gov/ct2/show/NCT03397121, accessed on 30 April 2022; ORION-10, https://clinicaltrials.gov/ct2/show/NCT03399370, accessed on 30 April 2022; ORION-11, https://clinicaltrials.gov/ct2/show/NCT03400800, accessed on 30 April 2022). On 22 December 2021, the FDA approved clinical use for the siRNA drug Leqvio (code 214012).

**(F)** A form of hereditary **Amyotrophic lateral sclerosis (ALS)** is caused by SOD1 mutations. The ASO “Tofersen” (BIIB067) is under investigation for ALS treatment and has been designed to mediate RNase-H-dependent degradation of SOD1 mRNA to reduce the synthesis of SOD1 protein. Mutations in SOD1 hit approximately 20% of all familial ALS cases and induce aggregation of misfolded SOD1. During the preparation of this review (June 2022), the European Network to Cure ALS officially announced the efficacy of tofersen in the treatment of ALS in the phase 3 clinical trial. Miller, T. et al. (2020) [45] published results about the Phase 1–2 trials of tofersen for SOD1 ALS. The authors reported that in adults with ALS due to SOD1 mutations, cerebral spine fluid (CSF) SOD1 concentrations decreased at the highest concentration of tofersen administered intrathecally over a period of 12 weeks. CSF pleocytosis occurred in some participants receiving tofersen. Lumbar-puncture-related adverse events were observed in most participants. (Funded by Biogen; ClinicalTrials.gov number, NCT02623699; https://clinicaltrials.gov/ct2/show/NCT02623699, accessed on 30 May 2022; EudraCT number, 2015-004098-33 https://www.clinicaltrialsregister.eu/ctr-search/search?query=2015-004098-33, accessed on 30 May 2022).

*(2). Splicing modifications of specific pre-RNAs by second- and third-generation modified ASOs: Nusinersen, Eteplirsen, and Milasen.*

**(A) Spinal muscular atrophy (SMA)** is a disease characterized by the loss of function of lower motor neurons and can be classified into four types according to the age of clinical outset: type 1 is the most severe form of SMA, it occurs before the age of 6 months and is associated with low expectancy of life and fundamental use of respiratory supports; type 2 occurs between the ages of 6 and 18 months; type 3 is divided in two forms—type 3a, which occurs before 3 years, and type 3b, which occurs after 3 years; type 4 occurs after 18 years and represents the adult form [46]. Pathogenesis of SMA is due to a mutation or deletion at the SMN1 gene encoding for the Survival Motor Neuron (SMN) protein that is crucial for the survival of motor neurons. The novel therapy of SMA focuses on another gene, SMN2 gene, which physiologically encodes mainly for a truncated SMN protein and on a smaller scale for a functional SMN protein. The difference between the two types of proteins is represented by the splicing of exon 7 that is skipped in SMN2 mRNA. Indeed, the regulation of the splicing of SMN2 mRNA can represent a therapeutic target because it is possible to increase the synthesis of a functional SMN2 protein by modifying the splicing process. “Nusinersen” (trade name Spinraza) produced by Biogen is a second-generation ASO with a 2′-O-methoxyethyl modification; it acts by regulating the splicing of SMN2 gene and its target is represented by the intronic splicing silencer whose activity is blocked by nusinersen. In this way, mature mRNA includes exon 7 and allows the production of a functional SMN protein [47]. Nusinersen was tested on a total of 149 infants, administered by intrathecal injection on days 1, 15, 29, and 64. Further maintenance doses were administered on days 183 and 302. In type 1 SMA, the drug induces an improvement of neurological function, expectancy of life, and a higher probability of survival without respiratory supports. Finally, the use of nusinersen cannot be considered as curative but it can improve the patient’s quality of life [48].

**(B) Duchenne muscular dystrophy (DMD)** is a primitive myopathy linked to X chromosome, in particular, to the DMD gene localized on region Xp21 that codifies for an important muscular protein called “dystrophin”. Men are generally affected and women are asymptomatic carriers. At birth, patients do not show any motor deficits, only high levels of creatine kinase. At the age of 2/3 years, some muscular deficits such as muscular weakness and difficulty to jump and run appear. At an age of about 10 years, they lose ambulation without use of crutches, and at age of 12 years they need to use a wheelchair. The DMD gene is composed of 79 exons and there are various mutations of this gene, the most common are deletions but there are also duplications and alterations of the reading frame such as the insertion of premature stop codon. In about 14% of DMD patients, there is a deletion of exons 49 and 50 and the introduction of a premature stop codon at exon 51 that determines, as a final effect, the lack of dystrophin’s production. It is possible to restore the reading frame by skipping exon 51; this determines the production of a dystrophin that it is shorter than the classical dystrophin but is functional. “Eteplirsen” (trade name “Exondys 51” produced by Sarepta) is a third-generation ASO, in particular, a **p**hosphorodiamidate **m**orpholino antisense **o**ligonucleotide (PMO, 5′-CTCCAACATCAAGGAAGATGGCATTTCTAG-3′) that is characterized by a neutral charge; its mechanism action is represented by the skipping of exon 51 (Figure 4) [49,50,51]. Eteplirsen is administered by intravenous infusion, and different clinical trials have been effectuated before the approval of FDA [52]. In these trials, efficacy of eteplirsen was demonstrated by the increase in dystrophin levels that are measured after muscle biopsies and evaluation of the effect on ambulation. The drug has been demonstrated to be able to delay the loss of ambulation and the deficit of other muscles such as respiratory muscles [51].

**(C)** An experimental, **tailored, splice-modulating, antisense oligonucleotide drug** called “Milasen” deserves to be mentioned. It was specifically designed to treat an 8-year-old girl (Mila is her name) suffering from a rare and fatal, progressive form of neurodegenerative disease leading to death by adolescence. J. Kim and coworkers designed [53], planned, and produced milasen by collaboration with a company in just 12 months from diagnosis to treatment. Milasen is a 22-nucleotide antisense oligonucleotide with the same structure and sugar chemistry modifications (phosphorothioate and 2′-O-methoxyethyl) as nusinersen to correct mis-splicing and restore normal (exon 6–exon 7) splicing and MFSD8 expression in the young patient. Dose–response analysis has indicated that its half-maximal potency is in the nanomolar range. RNA-seq from patient fibroblasts showed that milasen treatment more than tripled the amount of normal splicing in MFSD8 transcript. The experimental drug approved by the FDA inaugurates the new era of ultrapersonalized medicine, which will lead to rewriting all the rules, from experimentation to drug approval.

**Table 2:** List of siRNAs for cancer treatment.

Target	Drug	Cancer	Clinical Trial Identifier	References
p53	Teprasiran	Delayed graft function (DGF) in kidney transplantation	NCT02610283	[37,38]
TTR	Patisiran	Hereditary Transthyretin Amyloidosis (hATTR)	NCT01960348	[29,30,31]
Caspase 2	QPI1007	Nonarteritic ischemic optic neuropathy (NAION)	NCT01064505	[40]
KRASG12D	SigG12D-LODERs	Locally advanced pancreatic ductal adenocarcinoma LA-PDAC	NCT01676259	[54,55,56,57,58,59]
EphA2	DOPC nanoliposomalsiRNA (EPHARNA)	Advanced cancers	NCT01591356	[60,61,62]
Bcl2Like12 (Bcl2L12)	Spherical Nucleic Acids (SNA)	Glioblastoma multiforme	NCT03020017	[63]

## 4. Potential Molecular Targets for Transcript-Targeted Therapy in Cancer

### 4.1. Androgen Receptor (AR)

**Androgen Receptor (AR)** is a member of the nuclear receptors family; they are typical receptors of steroid hormones [64]. Androgen receptor plays a key role in prostate cancer that does not respond to castration therapy. This resistance is due to various alterations such as androgen receptor overexpression, point mutations, changes in androgen biosynthesis, and constitutive activation of AR [64]. These alterations make AR a critical therapeutic target and an ongoing clinical trial is aimed to evaluate a novel AR inhibitor in patients affected by castration-resistant prostate cancer. The inhibitor, called AZD5312 or ARRx, is a generation 2.5 of ASO that binds AR mRNA and inhibits the production of AR; the effect on tumor cells is represented by the inhibition of cellular growth and by the promotion of apoptosis (Table 3). The efficacy of this generation of ASO was evaluated in a preclinical study that focuses on the role of androgen receptor full-length (AR_FL_) and androgen receptor splice variants (AR_Vs_) [65]. ARRx is tested in combination with enzalutamide in a phase 1 and 2 ongoing clinical trial (ClinicalTrials.gov number: NCT03300505, https://clinicaltrials.gov/ct2/show/NCT03300505, accessed on 30 April 2022). Moreover, De Velasco et al. (2019) [66] demonstrated the improved efficacy of the use of generation 2.5 ASOs targeting the mouse AR in combination with the potent pan-AKT inhibitor AZD5363 in terms of prolonged survival in a clinically relevant mouse model of advanced castration-resistant prostate cancer (CRPC).

### 4.2. Breast Cancer Type 2 Susceptibility Protein (BRCA2)

**Breast cancer type 2 susceptibility protein (BRCA2)** is a tumor suppressor protein involved in the error-free repair of DNA double strand breaks (DSB) caused by environmental and medical radiation or generated during crossing over in meiosis. BRCA2 mutations (but also mutations of the related protein BRCA1) are associated with the increased risk of breast and ovarian cancer, as well as other types of cancer [67,68]. For BRCA-mutated ovarian cancer, FDA has approved the use of olaparib, an inhibitor of PARP-1, which is an enzyme involved in DNA single strand break (SSB) repair and in DNA replication [69,70]. The inhibition of PARP-1 caused by olaparib induces replication fork stalling resulting in double-strand break (DSB) that causes failure of replication and, subsequently, apoptosis unless homologous recombination repair (HRR) mechanisms occur [71,72]. This means that olaparib is effective only in HRR-deficient cells, while HRR-proficient cells are resistant [73,74]. Despite the therapeutic potential of PARP-1 inhibitors (such as olaparib), they can be used only for the treatment of tumors predominantly composed of HRR-deficient cells, since their use in a heterogeneous tumor cells population with a high rate of HRR-proficient cells can quickly lead to excessive growth of HRR-proficient clones and, therefore, to drug resistance [75,76]. One of the mechanisms that makes tumor cells HRR-proficient is BRCA2-reversion mutation [74]. A preclinical study showed that the use of an BRCA2-targeting antisense oligonucleotide, in combination with olaparib, sensitized numerous human cancer cell lines to this drug, thus increasing the incidence of chromosomal translocations and aneuploidies and preventing resistance to olaparib (and in general to PARP-1 inhibitors) in various tumor cell populations [77].

### 4.3. Clusterin

**Clusterin** is a chaperone protein that is part of the family of heat shock proteins [78]. It is an antiapoptotic protein that can interfere with BCL2 and NfkB. Several tumors present high levels of clusterin, facilitating the escape of programmed cell death [79]. In particular, prostate cancer shows overexpression of clusterin and, at the clinical level, resistance to androgen deprivation, chemotherapy, and radiotherapy [80,81]. Two phase 3 clinical trials, called SINERGY and AFFINITY, use Custirsen also called OGX-011, a second-generation 2′methoxyethyl-modified phosphorothioate ASO that binds clusterin mRNA in order to improve the sensitivity of cancer cells to antitumor therapies [82] (Table 3). Both trials select patients with metastatic castration-resistant prostate cancer. Previously, OGX-011 was tested in cell lines and in mice, demonstrating the ability to reduce clusterin expression and also to improve or restore chemosensitivity in both in vitro and in vivo models [83].

The aim of SINERGY trial is to compare survival of patients treated with custirsen, docetaxel, and prednisone. In the other arm, there are patients treated with docetaxel and prednisone but without custirsen. Results show that there is no significant improvement in the survival of patients treated with custirsen; only in patients with a poor prognosis did treatment with custirsen improve survival compared with treatment of docetaxel and prednisone alone. Another important point is the higher number of adverse drug reactions in patients treated with custirsen [84]. The AFFINITY trial attempts to value the improvement of survival in patients treated with custirsen, cabazitaxel, and prednisone compared with cabazitaxel and prednisone alone. Similar to the SINERGY trial, the AFFINITY trial also includes either patients with poor prognoses or general patients. The results are similar to those of the SINERGY trial and demonstrate that there is no significant improvement of survival of patients treated with custirsen. Moreover, in the AFFINITY trial, no improvement of survival was observed in poor prognosis patients, in contrast with the result of SINERGY [85]. A multinational, randomized, open-label study of custirsen in patients with advanced or metastatic (Stage IV) Non-Small-Cell Lung Cancer (https://www.clinicaltrials.gov/ct2/show/NCT01630733, accessed on 30 April 2022) has been planned between 2012 and 2017 but no results were posted for this study.

### 4.4. Epidermal Growth Factor Receptor (EGFR)

**Epidermal growth factor receptor (EGFR)** is a transmembrane protein—the main member of ErbB family proteins, which includes four structurally related receptor tyrosine kinases. EGFR binds specific ligands, such as epidermal growth factor (EGF) and transforming growth factor α (TGFα), initiating several signal transduction cascades involved in DNA synthesis and cell proliferation, principally MAPK, Akt, and JNK pathways (Table 3). These features suggest that mutations involving EGFR overexpression are associated with the development of a number of cancers [86]. EGFR has been identified as a therapeutical target of an antisense plasmid DNA (EGFR-AS) for the treatment of head and neck squamous cell carcinoma (HNSCC) [87,88]. Currently, the treatment of choice in Europe and USA for Head and Neck Squamous Cell Carcinoma (HNSCC), especially for elderly (>65 years old), frail, or unfit for cisplatin patients, consists in systemic administration of Cetuximab (a monoclonal antibody that inhibits EGFR) in combination with radiotherapy (RT) [89,90]. After checking the effective antitumor effects on preclinical HNSCC models, a phase 1 TRIAL (with a cohort of 11 patients) was carried out to verify whether systemic administration of Cetuximab RT combined with intratumoral injections of EGFR-AS effectively increased the antitumor effects of the current elective therapy, particularly at locoregional level. Indeed, locoregional failure remains the leading cause of death after cisplatin or Cetuximab treatment. The results confirmed the increased antitumor activity of the double inhibition of EGFR provided by Cetuximab and EGFR-AS associated to radiotherapy, as well as the good tolerance of the simultaneous administration of these two drugs’ controls [91]. Although this approach appears to be quite promising, a phase 2 trial is necessary to confirm the effective safety and efficacy of this combined treatment.

### 4.5. Eukaryotic Translation Initiation Factor 4E (eIF4E)

**Eukaryotic translation initiation factor 4E(eIF4E)** is a translation initiation factor involved in directing ribosomes to the 7-methyl-guanosine five-prime cap structure of mRNAs, an altered nucleotide on the 5′ of some transcripts that plays a key role in several cellular processes including mRNA stability and translational efficiency. It has been shown that eIF4E overexpression causes tumorigenic transformation in different cell lines and its expression is dysregulated in 30% of human cancers, such as cancer of the colon, lung, prostate, and breast [92]. For these features, EIF4E has been identified as a target of a second-generation antisense oligonucleotide called ISIS 183750 for the treatment of colorectal cancer (Table 3). In vitro experiments, aimed to verify the potential additional effects of a combined EIF4E ASO-irinotecan treatment, have been performed in colorectal cell lines. Based on these results, a clinical trial evaluating ISIS 183750 in patients with irinotecan-refractory colorectal cancer was conducted. The results showed that, despite the proven penetrance of ISIS 183750 into the target cells and elicitation of the pharmacodynamic effect of EIF4E inhibition, the combination of ISIS 183750 with irinotecan did not lead to objective results in patients with irinotecan-refractory colorectal cancer, perhaps due to a vast stromal binding of the ASO that may have caused a low cellular uptake. Nevertheless, the clinical activity of the combined therapy was in part demonstrated by stabilization in a subset of patients [93].

### 4.6. FoxP3

**FoxP3** is a transcription factor expressed by regulatory T cells (Treg), an important subset of CD4 T cells, that have a suppressive role in immune system regulation [94]. FoxP3 determines the production of CTLA4 that binds B7, expressed by antigen-presenting cells (APC); in this way, CTLA4 blocks the interaction between B7 and CD28 that is important for T-cell activation. Another mechanism involved in the inhibition of the activity of T cells is represented by the endocytosis of B7, an event leading to low response of T cells. High levels of FoxP3 have been detected in several cancers. suggesting that FoxP3 represents a suitable target for treatment. A preclinical study exploited a second-generation ASO, 2′-OMe-PS-ASO, in order to silence FoxP3 in B16 melanoma cells (Table 3). In particular, the aim of the research was the efficacy assessment of FoxP3 silencing associated with therapeutic vaccination. Furthermore, ASO is compared with polypurine reverse Hoogsteen hairpins (PPRHs). Results show that ASO penetrates in the cells better than PPRHs, displays a better silencing activity, and requires minor doses to obtain the therapeutic effect. The combination of ASO and vaccination was able to delay tumor growth and improve survival in mice. The results suggest that the synergic activity of vaccination and FoxP3-inhibitor ASO can be a novel strategy for cancer treatment [95].

### 4.7. Grb2

**Grb2** is a protein involved in signal transduction pathways linked to tyrosine kinase receptors and MAP kinases. Alterations of Grb2 protein can have a crucial role in cancer development. Two clinical trials focus on Grb2 overexpression in acute myeloid leukemia and in Philadelphia chromosome positive (Ph^+^) chronic myelogenous leukemia. In these tumors, overexpression of Grb2 is very important for cancer development and its inhibition could represent a novel strategy of treatment. In both trials, the drug used to reduce the level of Grb2 is the liposomal Grb2 ASO (L-Grb2)—code name BP1001—that prevents the synthesis of Grb2 protein (Table 3).

Use of this drug is supported by preclinical studies. In fact, the inhibition of Grb2 plays a central role in cell proliferation of leukemia patients [96]. Moreover, another study demonstrated the increased survival of mice treated with L-Grb2 compared with the liposomal control oligonucleotide [97].

The clinical trials use different combinations of BP1001 with standard drugs. The phase 2 clinical trial in acute myeloid leukemia evaluates the combination of BP1001 with venetoclax plus decitabine compared with the combination of BP1001 with decitabine. This trial is in recruiting status (ClinicalTrials.gov Identifier: NCT02781883, https://clinicaltrials.gov/ct2/show/NCT02781883, accessed on 30 April 2022).

### 4.8. KRAS

**KRAS** was first identified as a viral oncogene in the Kirsten RAt **S**arcoma virus [98]. Similar to the other member of the ras family, it is a GTPase that acts as a molecular on/off switch in many signal transduction pathways controlling cell proliferation. KRAS in human genome acts as a proto-oncogene whose mutations are implicated in several malignancies, about 20% in all human cancers, including lung adenocarcinoma, ductal carcinoma of the pancreas, and colorectal cancer [99,100,101]. Constrained ethyl residue (cEt) KRAS antisense oligonucleotide AZD4785 is an antisense oligonucleotide that contains 2′-4′ ethyl residue and targets with high affinity to both wild-type and mutated KRAS mRNAs, resulting in inhibition of downstream effector pathways and antiproliferative effects in cancer cells including lung and colon cancer cell lines [102,103] (Table 3). A phase I, open label, multicentre, dose escalation study was conducted to verify safety, maximum tolerated dose (MTD), and pharmacokinetics of AZD4785 (IV administered) in patients with KRAS-driven advanced solid tumors (ClinicalTrials.gov Identifier: NCT03101839, https://clinicaltrials.gov/ct2/show/NCT03101839, accessed on 30 April 2022). The results of this study are not yet available. Recently, a potent and selective antisense oligonucleotide AZD4785 has been chosen to target and downregulate all KRAS isoforms and demonstrated its ability to silence KRAS and to inhibit multiple myeloma-tumor-bearing KRAS mutations [104].

### 4.9. Hypoxia-Inducible Factor-1alpha (HIF-1alpha)

**Hypoxia-inducible factor-1alpha (HIF-1alpha)**, normally activated in response to hypoxia-induced stress, is a key transcription regulator of a large number of genes important in cellular adaptation to low-oxygen conditions, including angiogenesis, cell proliferation, apoptosis, and cell invasion [105,106]. A synthetic antisense oligodeoxynucleotide targeting hypoxia-inducible factor-1alpha (HIF-1alpha) with potential antineoplastic activity has been designed (EZN-2968, Table 3) in order to block HIF-1alpha protein expression, resulting in the inhibition of angiogenesis, the inhibition of tumor cell proliferation, and apoptosis. Nevertheless, due to its potential systemic side effects, EZN2968 is partially used in the clinic. For this reason, Zhang et al. (2021) [107] proposed and generated a conditional ASO able to inhibit HIF-1alpha in cells expressing the target miRNA as a hepatocyte-specific miRNA, miR-122, via a toehold-exchange reaction [107].

### 4.10. Heat Shock Protein27 (Hsp27)

**Heat shock protein27 (Hsp27)** is a chaperone protein and a member of heat shock proteins. This family of proteins has numerous functions such as chaperone activity, regulation of apoptosis, cell differentiation, and signal transduction [108]. Hsp27 can determine the growth and metastasis of cancer cells and also resistance to therapeutic agents. Activation of heat shock proteins can be due to cell stressors such as hyperthermia, oxidative stress, and radiation. Hsp27, in particular, can be activated by the cytotoxic effects of chemotherapy. Preclinical evidences show the role of Hsp27 in cancer, such as its role in bladder cancer cell. One study used OGX-427, called apatorsen, to knock down Hsp27 expression both in cancer cell lines and in mice (Table 3). Overexpression of Hsp27 increases cell growth and reduces chemosensitivity; on the other hand, OGX-427 reduces cell growth and sensitizes cancer cells to paclitaxel [109]. Taking into account these results and the high expression of Hsp27 in various types of cancer, two clinical trials—the Borealis-1 and the RAINIER trial—were designed to demonstrate the efficacy of a second-generation Hsp27ASO called apatorsen in cancer treatment.

Borealis-1 is a phase 2 trial that evaluates the efficacy of apatorsen in combination with gemcitabine and cisplatin compared with gemcitabine and cisplatin plus placebo, in patients affected by advanced urothelial cancer. Outcomes of this trial demonstrate a failure of apatorsen in improvement of survival. However, apatorsen shows a positive effect on survival in patients with poor prognosis. Patients with poor prognosis express higher levels of Hsp27 and circulating tumor cells (CTC) than patients with better prognosis; so, Hsp27 could be used as a biomarker to indicate patients that could be treated with apatorsen [110]. In the clinical study “Borealis-2”, Rosenberg et al. (2018) [111] reported the efficacy and safety of apatorsen in combination with docetaxel compared with docetaxel alone in patients with metastatic urothelial carcinoma previously treated with platinum-based chemotherapy. The randomized, controlled phase II trial with a primary end point of overall survival should provide strong elements to decide whether to move forward with a phase III trial.

The RAINIER trial is a phase 2 trial (ClinicalTrials.gov Identifier: NCT01844817, https://clinicaltrials.gov/ct2/show/NCT01844817, accessed on 20 June 2022) that compared apatorsen or placebo both combined with gemcitabine and nab-paclitaxel. Patients were affected by metastatic pancreatic cancer. The use of apatorsen did not improve overall survival (OS) of the patients, although a trend toward prolonged OS was observed in patients with high serum level of Hsp27 [112].

### 4.11. MicroRNAs

**MicroRNAs** (miRNAs) are a class of short (19–25 nt) noncoding RNAs that play an important role in several regulatory processes (such as proliferation, differentiation, metabolism, and apoptosis) by binding 3′-UTR of mRNAs. Aberrant expression of miRNAs is associated with a wide range of human diseases, including cancer; for this reason, these oligonucleotides are called oncoMir [113]. “SNAIL” is a protein involved in the invasiveness, sphere-forming ability, and induction of epithelial-mesenchymal transition (EMT) in ovarian cancer cells, an important step involved in metastasis formation [114]. Two microRNAs, miR-137 and miR-34a, have been identified: they can bind the 3′-UTR of SNAIL mRNA, reduce its expression, and reduce all the effects of SNAIL overexpression in ovarian cancer [115]. These two microRNAs could represent a valid candidate for the development of therapeutical alternatives for this type of cancer. “TRAIL” is a cytokine that could play an important therapeutic role in the treatment of various types of cancer, as it is able to induce apoptosis without damaging nearby tissues [116,117]. However, its application is limited because often cancer cells, and above all the cancer stem cells (CSCs), develop resistance to this type of treatment [118]. A preclinical study was conducted, which demonstrated that upregulation of miR25 in liver cancer stem cells (LCSs) induces resistance to TRAIL-induced apoptosis and concluded that knocking-down this microRNA, through its antisense oligonucleotide, increases the sensitivity of LCSs to TRAIL action [119]. In light of these evidences, downregulation of mir25 by its ASO could represent an interesting future therapeutic alternative for the treatment of several types of tumor (Table 3).

### 4.12. Ribonucleotide Reductase (RNR)

**Ribonucleotide reductase (RNR)** is a primary enzyme involved in synthesis of DNA. It consists of a large subunit (RNR1) and a small subunit (RNR2) associated to form a heterodimeric tetramer whose function is to remove the 2′-hydroxyl group of the ribose ring of nucleoside diphosphates catalyzing the formation of deoxyribonucleotides from ribonucleotides [120]. The inhibition of RNR causes the block of DNA synthesis and cell apoptosis [121]. GTI-2040 is a novel 20-mer phosphorothioate oligonucleotide that targets the mRNA of R2 subunit of RNR, preventing its interaction with ribosomes, spliceosomes, and other proteins involved in translation, as well as facilitating RNase-H-mediated degradation of RNA/DNA hybrids and inhibiting DNA transcription by forming a DNA triplex (Table 3). A preclinical study demonstrated a significant reduction in R2 mRNA and protein levels in numerous tumor cell lines, such as melanoma, colon, breast, pancreatic, ovarian, lung, and glioblastoma. Based on the results of preclinical studies, a phase I trial was conducted in which the effectiveness of GT-2040 (administered by continuous intravenous infusion) was tested in combination with gemcitabine hydrochloride in a cohort of 16 patients with advanced solid tumors. Gemcitabine is a nucleoside analogue that inhibits RNR, blocking the synthesis of DNA and the progression of cells through the G1/S-phase boundary. Due to these characteristics, it possesses antitumor activity (both in vitro and vivo) and its use has been approved for the treatment of various solid tumors, including pancreatic, bladder, nonsmall lung, and ovarian cancers. The study showed that the combined treatment of GT-2040 and gemcitabine, despite having an acceptable safety profile in pretreated patients (the most common adverse events being fatigue, nausea, vomiting, diarrhea, and anorexia), does not present a relevant antitumor activity. However, a partial clinical activity was demonstrated as several patients had prolonged stable disease [122].

### 4.13. Signal Transducer and Activator of Transcription 3 (STAT3)

**Signal transducer and activator of transcription 3 (STAT3)** is a member of the STAT protein family that includes seven different types of STAT protein. Mainly, STAT3 plays a central role in the transduction of the signal by different receptors acting as a transcription factor but it is also important in the regulation of immune system and, through these mechanisms, it can contribute to the development of cancer cells. It is known that inflammation can promote cancer cells and, obviously, transcription factors are crucial for tumor growth [123].

Based on these evidences, STAT3 has been chosen as a target in novel cancer treatment strategies: a drug designed to target STAT3 is AZD9150, called Danvatirsen, a generation 2.5 ASO with a constrained-ethyl group produced by IONIS^TM^ in partnership with AstraZeneca (Table 3). The efficacy and safety of AZD9150 were verified by different preclinical studies. Toxicology and pharmacokinetics were tested in mice and cynomolgus monkeys and the results showed a safety profile as in 2nd generation ASOs [124]. Furthermore, another preclinical work demonstrated the efficacy of AZD9150 to reduce levels of STAT3 mRNA and protein. In this way, the drug could determine an antitumor effect in both in vitro and in vivo models [125].

AZD9150 was also evaluated in neuroblastoma cells, where it was shown to be able to reduce STAT3 levels; this inhibition of STAT3 determines slow growth and also reduces the number of cellular colonies. Other effects include a possible alteration in tumor initiation and an increase in tumor cell chemosensitivity [126].

Another work focused on STAT3 inhibition in castration-resistant prostate cancer. In particular, inhibition of STAT3 was tested in combination with the stimulation of toll-like receptor 9; a STAT3 ASO was conjugated with a TLR9 agonist (a CpG oligonucleotide). Preclinical study has shown efficacy of these CpG-STAT3 ASOs to potentiate immune system activity against cancer cells, thanks to the reduction in tumor immune tolerance [127].

Furthermore, AZD9150 was also tested in a phase 1 clinical trial, started in July 2016 and completed in February 2019, aimed at finding the maximum tolerated dose and information about toxicity, pharmacodynamics, and pharmacokinetic profiles. The trial selected patients with diffuse large B-cell lymphoma, and drugs administered were combinations of MEDI4736 (durvalumab) and AZD9150, MEDI4736 as monotherapy, or MEDI4736 in combination with tremelimumab. No results have been yet communicated (ClinicalTrials.gov Identifier: NCT02549651, https://clinicaltrials.gov/ct2/show/NCT02549651, accessed on 30 April 2022).

**Table 3:** List containing the gene targets, their proposed transcriptional drugs, and the tumor where the drug potentially acts; the associated Clinical Trial Identifier code is reported if assigned.

Target	Drug	Cancer	Clinical Trial Identifier	References
**Androgen Receptor (AR)**	AZD5312 (ARRx)	Castration-resistant prostate cancer	NCT03300505	[64,65,66]
**BRCA2**	BRCA2-ASO	Ovarian cancer	---	[68,69,70,71,72,73,74,76,77]
**Clusterin**	OGX-011 (Custirsen)	Metastatic castration-resistant prostate cancer		[82,83,84,85]
**EGFR**	EGFR ASO	Head and neck squamous cell carcinoma		[87,88,89,90,91]
**EIF4E**	ISIS183750	Colorectal cancer	---	[93]
**FoxP3**	2′-OMe-PS-ASO	B16 melanoma cells		[95]
**Grb2**	BP1001 (L-Grb2)	Acute Myeloid Leukemia and (Ph+)chronic myelogenous leukemia	NCT02781883 NCT02923986	[96,97]
**KRAS**	AZD4785	Advanced solid tumors	NCT03101839	[99,100,101,102,103,104]
**HIF-1α**	ASO EZN-2968			[107]
**Hsp27**	OGX-427 (Apatorsen)	Advanced urothelial cancer and Metastatic pancreatic cancer		[109,110,111,112]
**miRNA**	miRNA25 ASO	Liver cancer cells	---	[113,114,115,116,117,118,119]
**Ribonucleotide Reductase (RNR)**	GTI-2040	Advanced solid tumor	---	[120,121,122]
**STAT3**	AZD9150 (Danvatirsen)	Diffuse large B-cell lymphoma	NCT02549651	[124,125,126,127]

## 5. siRNA Targets in Cancer

In this section, we will summarize novel findings about use of siRNA in cancer treatment. Several siRNAs were tested in preclinical models in vivo and in vitro, and some clinical trials started to demonstrate the safety, efficacy, and possible use of siRNA in novel anticancer therapeutic strategies. However, improvement of the delivery system is considered crucial to obtain a significant effect. A rather extensive description on the studies performed until 2017 was reported by Chen et al. [128]. In this review, we focus on more recent findings about siRNA in cancer therapy. In Table 2, we report the gene targets, their proposed siRNA, and the tumor where the drug potentially acts; the associated Clinical Trial Identifier code is reported if assigned.

### 5.1. Ephrin Type A Receptor 2 or Ephrin Receptor A2 (EphA2 Receptor)

**Ephrin type A receptor 2 or Ephrin receptor A2 (EphA2 receptor)** is a tyrosine kinase receptor encoded by the EPHA2 gene. The ligand of this receptor is represented by ephrin A1 (encoded by the human gene *EFNA1*). This interaction between ephrin A1 and EphA2 receptor can drive different tumorigenic events such as cell proliferation, migration, and angiogenesis. These effects are the likely explanation for the observed overexpression of EphA2 receptor in different cancers [60].

Some preclinical studies have targeted EPHA2 mRNA using a 1,2-Dioleoyl-sn-GlyceroPhosphatidylcholine (DOPC) nanoliposomal siRNA, also called EPHARNA, in order to decrease expression of EphA2 receptor (Figure 1, Table 2). First of all, efficacy of this drug was demonstrated by studies in cell lines and in mice. EphA2 receptor siRNA can reduce transcript levels in in vitro and in vivo models. The decrease in tumor growth is more significant when EphA2 receptor siRNA is administered in combination with paclitaxel. A decrease in microvascular density is also observed, confirming an antiangiogenic role of EphA2 receptor siRNA [61].

Preclinical studies demonstrate a safety profile of EPHARNA in mice and nonhuman primates [62]. A phase 1 clinical trial is ongoing (https://clinicaltrials.gov/ct2/show/NCT01591356, accessed on 30 May 2022), with the aim to evaluate the use of DOPC-EphA2 siRNA in advanced cancers. The clinical trial started in 2015 and is still recruiting. The result of this trial could provide some important clinical data.

### 5.2. KRAS

**KRAS** was first identified as a viral oncogene in the Kirsten Rat Sarcoma virus [98]. KRAS mutations (particularly in codons 12, 13, and 16) are present in almost all pancreatic adenocarcinomas. The importance of KRAS in cell signaling mechanisms makes it a potential target for the development of therapeutic alternatives for the treatment of this disease. After the failure in the development of inhibitors of posttranslational farnesylation (FTI), which showed no clinical activity [54,129], and specific ASOs, which showed a low specificity since the wild-type and mutated KRAS differ only in one codon [55,56], attention was paid to the development of therapeutic solutions based on RNAi, which show extraordinary sequence specificity.

A preclinical study conducted on Panc-1 and MiaPaca-2 cell lines, two of the most common human pancreatic cancer cell lines, showed that KRAS RNAi-induced knocking-down induces changes in the malignant phenotype: both cell lines showed reduced proliferation and migration capacity and a considerable reduction in angiogenic potential. This experimental evidence justified continuation of the research activity in the development of RNAi-based therapeutic solutions for the treatment of pancreatic cancer [57].

The first-developed drug that targets KRAS is SigG12D-LODERs. SigG12D-LODERs is a polymeric matrix containing siRNAs that target the mutated KRAS oncogene, specifically KRASG12D, with high specificity and proven antitumor activity that consists in inhibiting KRAS translation with potential blocking effects of tumor growth. LODER^TM^ (local drug EleuteR) technology (developed and marketed by Silenseed) represents an innovative delivery platform that allows the insertion of RNAi-based drugs directly into the core of solid tumors using a standard endoscope ultrasound (EUS) biopsy procedure (Table 2). Furthermore, LODER protects siRNAs from degradation and guarantees their action for very long periods of time (few months or more).

An open-label phase I study was conducted on a cohort of patients with nonresectable locally advanced pancreatic ductal adenocarcinoma (LA-PDAC), in which a single dose of SigG12D-LODERs was administered with a standard EUS procedure, combined with gemcitabine given on a weekly basis. The study showed an excellent safety and tolerance profile, as well as the stabilization of the disease in a group of patients. These evidences led to the implementation of a phase II study [58].

A phase II study (ClinicalTrials.gov Identifier: NCT01676259, https://clinicaltrials.gov/ct2/show/NCT01676259, accessed on 30 April 2022), still in the recruitment phase, foresees the administration of 2.8 mg of SigG12D-LODERs in 12-week cycles to patients with unresectable LA-PADC combined with a classic chemotherapy process (Gemcitabine + nab-Paclitaxel). This study will employ a cohort of 80 people divided into two arms: one arm receives the combined therapy, the other only the chemotherapeutic treatment.

### 5.3. A Clinical Study of RNA Interference Based on the Use of “Spherical Nucleic Acids (SNA)”

A clinical study of RNA interference based on the use of “**Spherical Nucleic Acids (SNA)**” arranged on the surface of small, spherical, gold nanoparticles conjugated with radially oriented and densely packed siRNA oligonucleotides for the GBM oncogene “Bcl2Like12” (Bcl2L12) has been recently published (Table 2) [127]. In the paper, the effects of NU-0129 on Bcl2L12 have been evaluated. The Bcl2L12 gene expressed in glioblastoma multiforme is associated with tumor growth and its expression blocks apoptosis in tumor cells promoting tumor growth. Researchers think that targeting the Bcl2L12 gene with NU-0129 will help stop cancer cells. This is a first-in-human trial to determine the safety of NU-0129 that is able to cross the blood–brain barrier. The clinical study demonstrated that NU-0129 uptake into glioma cells correlated with significant underexpression of tumor-associated Bcl2L12 protein, as shown by comparison of NU-0129-treated recurrent vs. matched primary untreated tumor. The study supports that SNA nanoconjugates is a brain-penetrant precision medicine approach for the systemic treatment of GBM [59] (https://clinicaltrials.gov/ct2/show/NCT03020017, accessed on 30 April 2022).

## 6. Conclusions

The use of ASO- and siRNA-based therapeutics for the treatment of a range of genetic diseases is an established fact. It is likely that the improvement of delivery vectors and chemical formulations will translate into an improvement in clinical efficacy. On the contrary, the use of this kind of therapeutics is still an area of active investigation for cancer treatment. The achievement of clinical utility will probably require several efforts in order to define molecular targets for specific tumor subtypes and to design selective delivery procedures [63]. In particular, the simultaneous action on different endogenous targets is one of the advantages offered by these therapeutics. Indeed, it is possible to combine nucleic acids with different sequences in the same vector [130]. Network-based strategies and combined multiple silencing approaches provide an alternative tool to arrest cancer proliferation. It has been demonstrated in breast cancer cell lines that silencing of five key upregulated transcripts dramatically changes cell survival and migration [131]. In this sense, overexpression of genes encoding “eukaryotic Initiation Factors” or “Cleavage and Polyadenylation of Pre-mRNA” factors have been associated with colorectal tumors. These transcripts represent good candidates for transcript-targeted therapy [132,133,134]. Although these approaches have been already applied in preclinical models, they have not yet passed all the steps necessary for human investigations. Indeed, no clinical trials have been started exploiting the combinatorial silencing of multiple cancer targets [135]. Further studies are still needed to support the efficacy of combinatorial silencing.

## Data Availability

Not applicable.

## Abbreviations

**2-MOE**	2-O-methoxyethyl
**2-O-MCE**	2-O-methylcarbamoylethyl
**2-OME**	2-O-methyl
**AKI**	Acute kidney injury
**APOC2**	Apolipoprotein C2
**ApoE**	Apolipoprotein E
**AR**	Androgen Receptor
**ASO**	Antisense Oligonucleotide
**BAL**	Bronchoalveolar lavage
**BRCA**	Breast Cancer susceptibility protein
**CPP**	Conjugation with cellular Penetrating Peptide or using liposomal structure
**DGF**	Delayed Graft Function
**DSB**	Double Strand Break
**DSPC**	Phospholipid Distearoylphosphatidylcholine
**dsRNA**	Double-Stranded RNA
**EGFR**	Epidermal Growth Factor Receptor
**eIF4E**	Eukaryotic translation initiation factor 4E
**FCS**	Familial Chylomicronemia Syndrome
**Gapmer**	A central phosphorothioate oligonucleotide between two chemical sequences containing modified sugars.
**hATTR**	Hereditary **T**rans**t**hy**r**etin **A**myloidosis
**HIF-1alpha**	Hypoxia-inducible factor-1alpha
**Hsp27**	Heat shock protein27
**LDLR**	Lipoprotein cholesterol receptor
**LNP**	Lipid nanoparticle
**LPL**	Lipoprotein Lipase
**miRNA**	MicroRNAs
**NAION**	Nonarteritic ischemic optic neuropathy
**PMO**	Phosphorodiamidate Morpholino Oligonucleotide
**PNA**	Peptide Nucleic Acid
**PS ASO**	Phosphorothioate ASO
**RNAi**	RNA interference or RNA silencing
**RNR**	Ribonucleotide Reductase
**siRNA**	Short Interfering RNAs
**SMN**	Survival Motor Neuron protein
**SSB**	Single Strand Break
